# Chemotherapy-Induced Changes in Plasma Amino Acids and Lipid Oxidation of Resected Patients with Colorectal Cancer: A Background for Future Studies

**DOI:** 10.3390/ijms25105300

**Published:** 2024-05-13

**Authors:** Roberto Aquilani, Silvia Brugnatelli, Roberto Maestri, Paolo Iadarola, Salvatore Corallo, Anna Pagani, Francesco Serra, Anna Bellini, Daniela Buonocore, Maurizia Dossena, Federica Boschi, Manuela Verri

**Affiliations:** 1 Department of Biology and Biotechnology “Lazzaro Spallanzani”, University of Pavia, 27100 Pavia, Italy; dottore.aquilani@gmail.com (R.A.); paolo.iadarola@unipv.it (P.I.); anna.bellini03@universitadipavia.it (A.B.); daniela.buonocore@unipv.it (D.B.); maurizia.dossena@unipv.it (M.D.); 2 Medical Oncology Unit, Fondazione IRCCS Policlinico San Matteo, 27100 Pavia, Italy; s.brugnatelli@yahoo.it (S.B.); s.corallo@smatteo.pv.it (S.C.); A.Pagani@smatteo.pv.it (A.P.); francesco.serra03@universitadipavia.it (F.S.); 3 Department of Biomedical Engineering of the Montescano Institute, Istituti Clinici Scientifici Maugeri IRCCS, 27040 Montescano, Italy; roberto.maestri@icsmaugeri.it; 4 Department of Drug Sciences, University of Pavia, 27100 Pavia, Italy; federica.boschi@unipv.it

**Keywords:** colorectal cancer, FOLFOX and XELOX therapy, primary tumor location, plasma amino acid changes, plasma malondialdehyde changes, potential clinical implications

## Abstract

Previous studies have documented that FOLFOX and XELOX therapies negatively impact the metabolism of skeletal muscle and extra-muscle districts. This pilot study tested whether three-month FOLFOX or XELOX therapy produced changes in plasma amino acid levels (PAAL) (an estimation of whole-body amino acid metabolism) and in plasma levels of malondialdehyde (MDA), a marker of lipid hyper oxidation. Fourteen ambulatory, resected patients with colorectal cancer scheduled to receive FOLFOX (n = 9) or XELOX (n = 5) therapy, after overnight fasting, underwent peripheral venous blood sampling, to determine PAAL and MDA before, during, and at the end of three-month therapy. Fifteen healthy matched subjects (controls) only underwent measures of PAAL at baseline. The results showed changes in 87.5% of plasma essential amino acids (EAAs) and 38.4% of non-EAAs in patients treated with FOLFOX or XELOX. These changes in EAAs occurred in two opposite directions: EAAs decreased with FOLFOX and increased or did not decrease with XELOX (interactions: from *p* = 0.034 to *p* = 0.003). Baseline plasma MDA levels in both FOLFOX and XELOX patients were above the normal range of values, and increased, albeit not significantly, during therapy. In conclusion, three-month FOLFOX or XELOX therapy affected plasma EAAs differently but not the baseline MDA levels, which were already high.

## 1. Introduction

The combination of the chemotherapeutic agents 5-fluorouracil (5-FU) and oxaliplatin (OXAL) is the standard adjuvant treatment for patients with stage III colon cancer (CRC) [1]. Unfortunately, despite the improvement in disease-free survival and overall survival associated with their use as an adjuvant treatment, these drugs possess a number of side effects.

A total of 15 to 20% of patients on 5-FU [2] experience adverse events [3] such as myelosuppression, gastrointestinal and central nervous system toxicity [4,5], and cardiotoxicity [6].

The main adverse effects of OXAL, which may persist over time after treatment cessation [7], are gastrointestinal disturbances (nausea, vomiting, and diarrhea), peripheral sensory neuropathy [8,9], and acute thrombocytopenia [10]. Chemotherapy (CMT) toxicity often leads the physician to reduce/discontinue/interrupt the administration of the drug(s).

Like the other chemotherapeutic drugs, 5-FU and OXAL also elicit adverse effects on skeletal musculature [11] causing muscle dysfunction, weakness, fatigue, and atrophy. CMT-induced myopathy was previously described in experimental animals to whom FOLFOX therapy (containing 5-FU+OXAL+leucovorin) was provided [12,13,14,15,16,17].

In the current study, we hypothesized that one of the consequences of 5-FU and OXAL toxicities on muscle metabolism could be perturbations in muscle amino acid (AA) metabolism. These could derive from a higher rate of catabolic over anabolic processes, thus being indirectly reflected in changes in plasma AA levels (PAALs). This hypothesis was based on the following considerations. Firstly, skeletal muscle is the most important repository of proteins/AAs in the body [18]. Secondly, CMT may damage protein synthesis activities while enhancing protein catabolism. Indeed, protein synthesis could be impaired by several factors including CMT-induced inhibition of DNA and RNA synthesis, cancer-related and surgery-related insulin resistance [19], chronic inflammation [20,21,22,23], and mitochondrial dysfunction [24] resulting in lower energy production [25,26]. 

The rate of muscle protein catabolism may increase due to both CMT-induced cellular and mitochondrial-protein destruction [27] and the activation of muscle proteolytic pathways by an increase in oxidative stress [28,29,30,31,32].

Therefore, the aim of the current study was to document the time courses of PAALs over 3 months after the initiation of FOLFOX (containing 5-FU+OXAL+leucovorin) and XELOX (regimen formed by OXAL infusion + oral capecitabine, pro-drug of 5-FU) in a group of post-surgery patients with CRC. Moreover, we hypothesized that PAAL alterations may be more severe with the FOLFOX than with the XELOX regimen, for the following reasons. Firstly, while with FOLFOX the drugs are concomitantly infused, they are sequentially administered with XELOX. This could cause cells and tissues to be differently exposed to the drugs’ activity. Secondly, only 70–80% of oral capecitabine is bioavailable, thus making cells and tissues unexposed to the entire amount of the ingested drug [33].

Lastly, given the detrimental effects of reactive oxygen species (ROS) on mitochondria structure and function and the occurrence of ROS-activated proteolytic pathways, we also determined plasma levels of malondialdehyde (MDA), an established marker of cell lipid hyper oxidation [34].

The rationale of the current research was that the knowledge of possible CMT-induced PAALs, plasma lipid oxidation changes, and the potential underlying mechanisms could represent a framework for future studies addressing cancer immune tolerance [35,36,37,38] and CMT-induced adverse clinical factors [39].

## 2. Results

### 2.1. Baseline Patient Clinical Characteristics, PAAL and MDA (Time A, TA)

The study showed that patients treated with FOLFOX (FOLFOXs) and XELOX (XELOXs) had similar normal body weights (as BMI) and bio-humoral variables (Table 1).

In comparison to controls (CTR; matched for age, sex, and body mass index), FOLFOXs displayed more PAAL differences than XELOXs (Table 2), whose therapy showed that only the levels of histidine (*p* = 0.046) and tryptophan (*p* = 0.047) were higher. FOLFOXs had significantly increased plasma levels (Table 2) of histidine, tryptophan, phenylalanine, isoleucine, leucine, lysine, valine, methionine, essential AAs (EAAs), branched-chain AAs (BCAAs), and the EAA/total AA ratio (EAAs/TAAs). In FOLFOXs, the non-EAA/TAA ratio (NEAAs/TAAs) was lower.

FOLFOXs, XELOXs, and the CTR group had a similar (glutamine + alanine)/BCAA ratio.

With respect to gender differences, males and females showed similar ages, BMIs, bio-humoral variables (Table 3), and plasma AA profiles, with the exception of serine, histidine, cysteine, which were higher in female patients (Table 4). Moreover, the levels of plasma AAs normalized for the levels of serum creatinine, a surrogate of muscle mass tissue [40,41,42], were similar between males and females with the exception of serine/creatinine, histidine/creatinine, glycine/creatinine, threonine/creatinine, phenylalanine/creatinine, cysteine/creatinine which were higher in females (Table 5). 

No significant differences were found in PAALs between FOLFOXs and XELOXs.

The marker of lipid hyper oxidation (MDA) was higher than normal values (found in our laboratory) in both FOLFOXs and XELOXs.

MDA was significantly higher in FOLFOXs than in XELOXs (Table 1).

### 2.2. Time Courses of PAALs during the Three-Month CMT (Time A: Baseline → Time B: 28–32 Dys → Time C: 87–93 Days)

During CMT, both FOLFOX and XELOX groups maintained their baseline similar BMIs and serum creatinine levels (Table 6).

**Figure 1:**
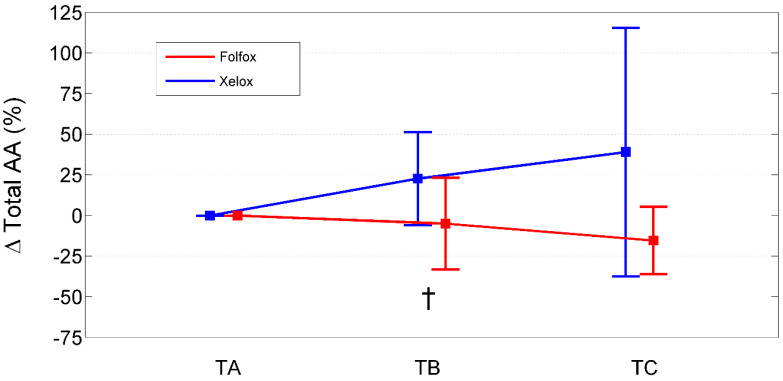
Percentage change from baseline (TA) of total amino acids in XELOXs and FOLFOXs at TB and TC. Data reported as mean (95% confidence interval). †: *p* = 0.03 for the comparison XELOX vs. FOLFOX at TB (Mann–Whitney U-test). Abbreviations: AAs, amino acids; TA, Time A; TB, Time B; TC, Time C.

**Figure 2:**
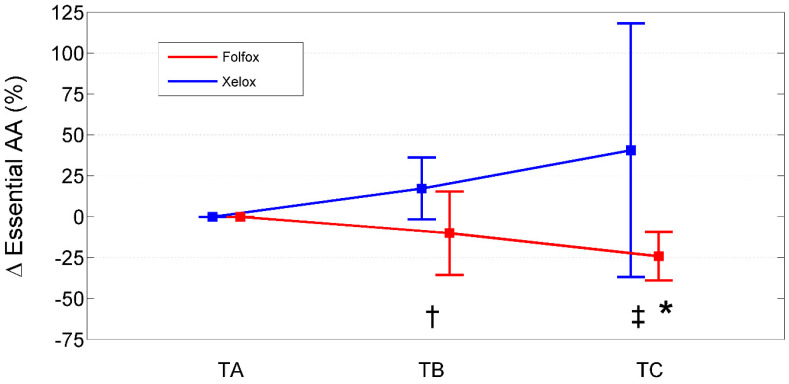
Percentage change from baseline (TA) of essential amino acids in XELOXs and FOLFOXs at TB and TC. Data reported as mean (95% confidence interval). †: *p* = 0.03 for the comparison XELOX vs. FOLFOX at TB (Mann–Whitney U-test). ‡: *p* = 0.003 for the comparison XELOX vs. FOLFOX at TC (Mann–Whitney U-test). *: *p* = 0.008 for the comparison FOLFOX TC vs. TA (Wilcoxon signed rank test). Abbreviations: AAs, amino acids; TA, Time A; TB, Time B; TC, Time C.

**Figure 3:**
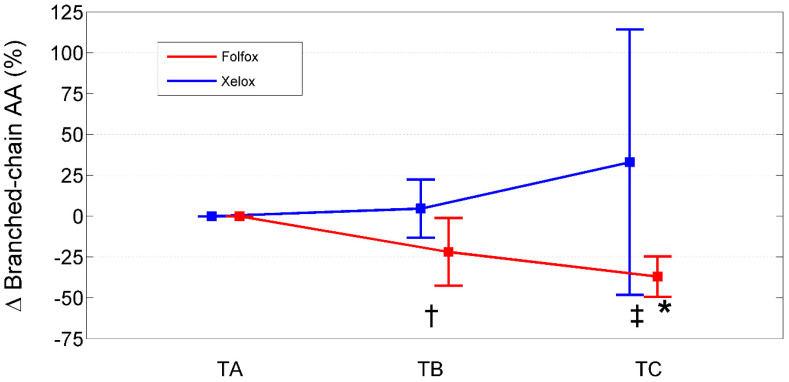
Percentage change from baseline (TA) of branched-chain amino acids in XELOXs and FOLFOXs at TB and TC. Data reported as mean (95% confidence interval). †: *p* = 0.04 for the comparison XELOX vs. FOLFOX at TB (Mann–Whitney U-test). ‡: *p* = 0.002 for the comparison XELOX vs. FOLFOX at TC (Mann–Whitney U-test). *: *p* = 0.008 for the comparison FOLFOX TC vs. TA (Wilcoxon signed rank test). Abbreviations: AAs, amino acids; TA, Time A; TB, Time B; TC, Time C.

Relative to baseline values (TA), PAALs changed in opposite directions depending on the chemotherapeutic agent used (Table 7; Figure 1, Figure 2 and Figure 3). Inter-group significant changes regarded were 87.5% of plasma EAAs and 38.4% of plasma NEAAs. In FOLFOXs, there were progressive decreases in serine, citrulline, alanine, arginine, tyrosine (partially), tryptophan, phenylalanine (partially), isoleucine, leucine, lysine, ornithine, valine, methionine, TAAs, EAAs, and BCAAs. By contrast, the XELOX regimen was associated with significant increases in the above-mentioned AAs.

Both FOLFOX and XELOX therapies induced similar increases in the (glutamine + alanine)/BCAA ratio.

The time courses of tyrosine/leucine ratios increased with both FOLFOX and XELOX therapies.

In relation to gender differences (six males and three females, all in the FOLFOX population), males and females underwent similar PAAL deteriorations with the exception of serine and isoleucine which were less and more impaired in females, respectively (Table 8).

### 2.3. Time Courses of Plasma MDA Levels during the Three-Month CMT

During CMT, plasma baseline MDA levels did not change significantly and remained high in both FOLFOXs and XELOXs, with a trend to higher absolute values in FOLFOXs than in XELOXs at time points A, B and C (Table 9).

In relation to gender differences, the MDA changes were similar between males and females (Table 8).

### 2.4. PAAL at the End of Three-Month CMT (Time C, TC)

Three months after therapy initiation (Table 10), FOLFOXs trended to reduced levels of asparagine, showed decreased serine, arginine, tryptophan, isoleucine, leucine, valine, ornithine, EAAs, BCAAs, and ratios of arginine/TAAs, BCAAs/EAAs, EAAs/TAAs and BCAAs/TAAs, but showed increased NEAAs/TAAs.

XELOXs trended to reduced levels of glutamine, histidine, glycine, alanine, phenylalanine, lysine, and the ratios of arginine/TAAs and BCAAs/EAAs.

The results showed that, compared to baseline values, at TC point while the (glutamine + alanine)/BCAA ratio significantly increased in FOLFOXs (*p* = 0.008), the increase for XELOXs was not significant.

### 2.5. Correlations between PAAL and Peripheral Blood White Cells 

**Figure 4:**
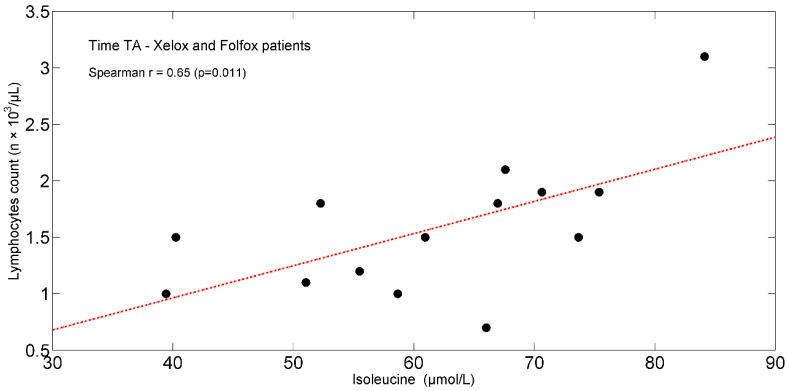
Scatterplot showing the relationship between isoleucine and lymphocyte count in XELOXs and FOLFOXs at TA. The linear regression line is also shown (red dash-dotted line). Abbreviations: TA, Time A.

**Figure 5:**
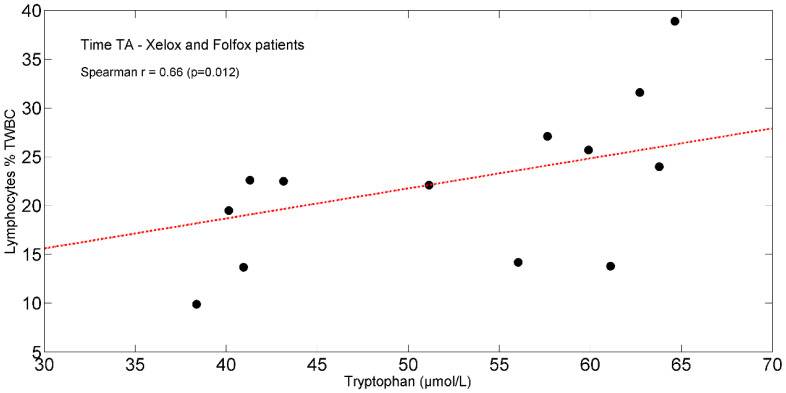
Scatterplot showing the relationship between tryptophan and lymphocytes (% of TWBC) in XELOX and FOLFOX patients at TA. The linear regression line is also shown (red dash-dotted line). Abbreviations: TA, Time A; TWBC, total white blood cells.

**Figure 6:**
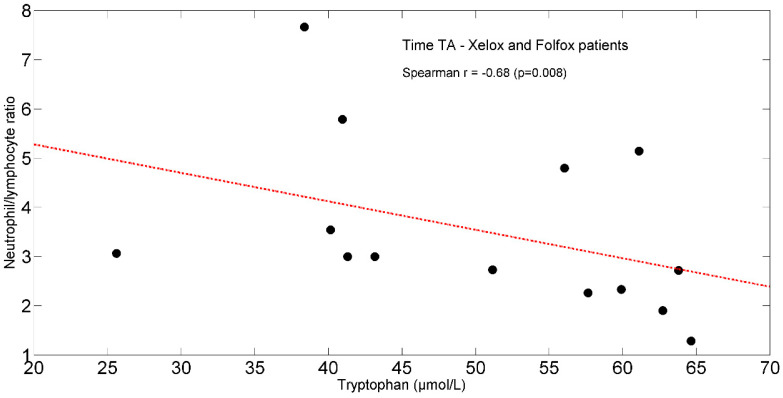
Scatterplot showing the relationship between tryptophan and neutrophil/lymphocyte ratio in XELOX and FOLFOX patients at TA. The linear regression line is also shown (red dash-dotted line). Abbreviations: TA, Time A.

In the entire population, at TA, the study found positive significant associations between plasma isoleucine (r = +0.65, *p* = 0.011) (Figure 4) and circulating lymphocytes. Moreover, tryptophan (r = +0.66, *p* = 0.012) (Figure 5) correlated with lymphocytes expressed as a percentage of total white blood cells (TWBC) but negatively with the neutrophil/lymphocyte ratio (r = −0.68, *p* = 0.008, Figure 6).

## 3. Discussion

The study shows that the state of oxidative stress displayed by resected patients with CRC pre-CMT did not change significantly during CMT. At baseline, oxidative stress in patients who were subsequently treated with FOLFOX was more significant than in those on XELOX, and this difference tended to be maintained during CMT.

The study found that three-month CMT changed the baseline PAAL. The changes occurred in two opposite directions, depending on the adopted chemotherapeutic agent and as hypothesized, the changes were worse with the FOLFOX than with the XELOX regimen. To explain this behavior, it was hypothesized that the different type of administration between the two therapeutic regimens and the reduced capecitabine bioavailability (70–80% of the ingested amount) [33] could lead to an impact of FOLFOX that was more accentuated than that of XELOX on cells/tissues. AA time courses during FOLFOX therapy were negative in relation to baseline values, whereas they were positive or neutral during XELOX. The changed AAs (mainly EAAs) were the same in both CMT regimens.

The worse time courses of AAs with FOLFOX were confirmed by the number of baseline AAs that were significantly reduced at the end of the third month, in comparison to the XELOX regimen.

Although differences in ethnicity, gender, age, diet, muscle mass, and cancer mutations can impact the PAAL, these factors likely were not determinant in explaining the different behavior of PAALs over time. Ethnicity did not influence PAAL, being that all patients were of Caucasic ethnicity (Italian subjects). Likely, age did not influence PAAL, as FOLFOX and XELOX populations had similar ages. Very probably, the habitual diets did not significantly change during treatment either in FOLFOXs or in XELOXs and were adequate to body needs during CMT both in terms of energy and protein provisions, as indicated by the maintenance of body weight and, indirectly, by the maintenance of normal serum creatinine levels. Indeed, protein malnutrition limits creatinine generation and results in low serum creatinine [43]. In addition, should the patients have adopted a protein-restricted diet, their plasma serine would have increased and not diminished as observed in our study [44]. The maintenance over time of similar serum creatinine levels between FOLFOXs and XELOXs also suggests that the two populations had no significant differences in skeletal mass tissue. There is a linear correlation between serum creatinine and total body muscle mass [40]. Indeed, serum creatinine levels are used as a surrogate of muscle mass [41,42]. Similar serum creatinine levels were found between male and female patients (however, the latter group was only three individuals), even if, as expected, the creatinine tended to be lower in females. Therefore, gender differences in muscle mass did not significantly influence PAALs. To support this, males and females showed similar ratios of AAs/creatinine.

It is highly probable that possible cancer mutations [45,46] had no effect in the overtime PAAL changes. Indeed, in the study patients, AA-consuming cancer biomass was eradicated, and this is in contrast with the post-surgery progressive PAAL changes.

Therefore, to interpret these preliminary results, we formulate plausible mechanisms that may serve as working hypotheses for future studies.

### 3.1. Potential Mechanisms Underlying PAAL

#### 3.1.1. Pre-CMT

Several factors may account for the high muscle release of AAs. They include patient pre-surgery- and cancer-related protein metabolism; the rate of the acute response to surgery with the development of insulin resistance and systemic inflammation; and possible post-surgical complications, and the time of and tolerant to the resumption of normal nutrition in FOLFOXs [47,48,49,50,51]. 

Compared to the CTR, FOLFOXs and XELOXs shared an increased muscle release of histidine. This could be due to high muscle consumption of histidine-containing carnosine, anserine, and β-alanine dipeptides, which have a role in the neutralization of ROS [52] and in buffering intracellular protons [52]. An elevated histidine level was more evident in female than in male patients. Of note, females had also higher plasma serine and cysteine concentrations. These differences were maintained even when normalized for creatinine. In addition, females showed higher plasma ratios of glycine/creatinine. All these AAs play important and synergistic roles in cell antioxidant capacity.

#### 3.1.2. During CMT

FOLFOX and XELOX exerted different toxicities on muscle and extra-muscular districts. As FOLFOX and XELOX contain the same molecule (apart from leucovorin in FOLFOX), the different AA time courses may be explained by the different route of drug administration (concomitantly infused with FOLFOX, sequentially administered with XELOX) and by the reduced bioavailability of ingested capecitabine (70–80% of the ingested dose) [53]. This contributes to explaining the wider pharmacokinetic variability in subjects on capecitabine than in those on 5-FU [33].

Therefore, the adverse effects on AA metabolism would be amplified with the simultaneous administration of 5-FU and OXAL [54], as it has been shown to happen in the heart [55,56].

While the present study was not planned to measure the body AA fluxes [57,58] that are necessary to understand AA inflow and outflow [59], we postulate that CMT-induced AA changes may be due to an excess of body EAA utilization, relative to patient EAA intake, and altered gut microbiota.

The AAs will only be discussed in relation to cancer.

- Hypotheses on mechanisms underpinning FOLFOX-induced AA time courses

In an attempt to explain AA changes induced by FOLFOX therapy, we speculate that two important mechanisms could be EAA overutilization in muscle tissue, with a consequent reduced EAA release, and in extra-muscular districts. 

As the venous blood was drawn from the patients’ antecubital vein, PAALs mainly express the tissue metabolism of skeletal muscle tissue [57]. 

Within the skeletal muscle, EAAs were presumably consumed for energy production in the mitochondria escaping FOLFOX toxicity, being that AAs are the only substrates activating the tricarboxylic acid cycle (TCA cycle) [60]. Two factors may support the pro-energetic use of BCAAs by muscle mitochondria. First, the skeletal muscle is the main site for BCAA metabolism and BCAAs are the most important fuel for skeletal muscle [61]. In this study, BCAA consumption for transaminase activities and energy production is indirectly suggested by the progressive increase in glutamine from TA to TC [61]. The glutamine increase is in contrast with alanine decrease, the other substrate formed by BCAA transamination. Presumably, alanine underwent significant transamination to pyruvate [62]. 

The preservation of glutamine generation is important to sustain patient immune responses [63] and reduce the risk of acute hyperammonemia encephalopathy in patients on fluoropyrimidine treatment, especially if sarcopenic and hypo hydrated [5]. 

It is unlikely that BCAA consumption occurred, inducing net protein synthesis within the muscle tissues, given that FOLFOX induces mitochondriopathy [11,30], impairment in energy generation (necessary for protein synthesis [64]), and hyperproduction of ROS [28,29,30,31,65], all factors promoting protein catabolism.

Increases in plasma phenylalanine and tyrosine [66] and in the tyrosine/leucine ratio [67] over time support the hypothesis of a net muscle hypercatabolic state. 

The metabolism of extra-muscular districts, such as the heart [68,69,70], the brain [71,72,73], the intestine [74], and all the organs that are characterized under physiological conditions by high oxidative metabolism and protein turnover, could have BCAA overconsumption during FOLFOX therapy [60,75]. We assume that the normally elevated myocardium BCAA consumption [60,68,76,77] might further increase following the impaired activity of the BCAA-α-ketoacid dehydrogenase complex caused by 5-FU treatment [78]. Several AAs, including the BCAA valine, are known to limit 5-FU cardiotoxicity [56]. In a rat model of colorectal cancer with liver metastasis, the dietary AA glycine prevented FOLFOX-induced myocardial toxicity by preserving the left ventricle performance and reducing both fibrosis and apoptosis [79].

5-FU-induced unbalanced microbiota [74,80,81,82,83,84] may contribute to intestinal inflammation (mucositis), leading to reduced circulating EAAs [62,85,86,87,88].

During FOLFOX therapy, the patients likely experienced a progressive intestinal dysfunction as indicated by the progressive reductions in plasma citrulline [89,90,91], partly responsible for low arginine formation [89,92]. 

Reduced arginine synthesis has previously been reported in cancer patients [58,93,94]. Low arginine in the study patients led to low ornithine levels [95].

Reduced synthesis and increased body utilization of serine are factors in the progressive decline in the plasma serine levels [96,97,98,99,100].

FOLFOX-induced intestinal inflammation and barrier dysfunction [80] may increase enterocyte uptake of dietary and plasma tryptophan [101], leading to reduced plasma levels of the AA. In addition, we cannot exclude that a tryptophan overutilization could occur for albumin synthesis [102] and in cells of the adaptive immune response [103]. This latter effect is also observed in the study patients by the baseline correlation between tryptophan and lymphocytes % TWBCs, and the neutrophil/lymphocyte ratio.

The importance of some EAAs in sustaining the adaptive immune response may be suggested by the baseline correlation between plasma isoleucine and peripheral blood lymphocyte counts [104].

The study shows that at the end of three-month FOLFOX therapy, the patients exhibited, independently of sex, an important deterioration in PAALs that may potentially have clinical implications (Table 11).

Table 12 summarizes some putative mechanisms underlying FOLFOX toxicity on body AA metabolism.

We are aware that the considerations made to explain FOLFOX effects on PAAL changes are speculative. Therefore, future, well-planned studies on a larger patient cohort are needed to verify the real importance of above mechanisms.

- Hypotheses on mechanisms underpinning XELOX-induced AA time courses

Attenuation of the metabolic alterations considered for FOLFOX toxicity might explain the AA time courses during the XELOX regimen, the time course differences between FOLFOX and XELOX regimens, and the attenuated deterioration of several AAs after three-month therapy. Attenuated metabolic alterations associated with XELOX may be shown by the maintenance over time of the (glutamine+alanine)/BCAA ratio [61], suggesting less perturbed muscle mitochondrial activity. In addition, a confirmation of attenuated metabolic alterations can be shown by the reduced baseline ratios of only BCAAs/EAAs and arginine/TAAs.

On the contrary, with FOLFOX, the study found a decrease in BCAAs/EAAs, arginine/TAAs, BCAAs/TAAs, EAAs/TAAs, arginine, and an increase in the NEAA/TAA ratio.

In any case, XELOX-induced AA changes represent a negative metabolic finding in that they may be an expression of prevalent muscle protein catabolism, as also suggested by increased muscle releases of phenylalanine, tyrosine, and by an increased plasma tyrosine/leucine ratio. 

However, XELOX-induced AA changes may have a clinical advantage in that they can be provided in adequate amounts to the various body compartments.

This factor may cause less muscle AA degradation [65,120,121] and more muscle AA release. 

One example of attenuated metabolic alterations during XELOX may be the time courses of citrulline [89,90,91] whose increase expresses reductions in intestinal inflammation and dysfunction, and hence less enterocyte AA overconsumption.

### 3.2. Potential Mechanisms Underlying Oxidative Stress Pre- and during CMT

The degree of oxidative stress pre-CMT was determinant in maintaining its level during CMT. This means that (at least as far as the patients in this study), patient cancer-related [122,123,124,125,126] and/or post-surgery inflammation-induced oxidative stress [127,128] were more important than CMT in causing ROS production.

No significant differences in lipid hyper oxidation were found between males and females.

Due to a lack of MDA information before surgery, the study cannot distinguish the importance of the contribution of cancer metabolism and of surgery inflammation to pre-CMT MDA.

The minor MDA changes during CMT were unexpected. However, we only partially measured cell oxidative stress, i.e., the attack of ROS mainly on membrane lipids, and we did not measure the markers of the free radical-driven attack of DNA, RNA, proteins, AAs, sugar, and phosphorus, which would likely have been more effective in capturing ROS-driven damage in both cell structure and macromolecules.

For example, the free radical attack of nitrogen compounds causes the generation of reactive nitrogen species (RNS), which interact with ROS [128].

Chronic oxidative stress largely contributes to muscle apoptosis and necrosis, which are both responsible for muscle atrophy [129,130,131].

It is reported that oxidative stress is an important mechanism involved in oxaliplatin-induced peripheral neuropathy [132,133,134,135,136]. 

In mice, the co-treatment OXAL + resveratrol, a potent antioxidant, not only prevented OXAL-induced neurotoxicity, but also attenuated gastrointestinal damage to the mucosa, crypts and muscle layer, leading to improved contractility and reduction in constipation [137].

In Table 11 are reported the potential negative impacts from altered plasma EAAs and lipid hyper oxidation on body systems. Thus, the co-presence of reduced EAA availability and increased ROS [11,30,138,139] may act additively/synergistically to muscle and organ/tissue loss of integrity and dysfunction. This may contribute to adverse events in the initial phase of CMT and even more when CMT duration lasts for more than three months [140]. 

### 3.3. Limitations

The present research has several limitations, requiring well-planned prospective investigations to be resolved. The first limitation is the limited sample size, which is too small to draw any definitive conclusions. Given that, to our knowledge, the time courses of AA changes during treatment are totally unknown, it was impossible to calculate the appropriate sample size for this study. Therefore, the results of this investigation must be considered preliminary and should be confirmed by studies involving a larger number of patients, stratified on the specific disorder. 

The absence of AA flux measurements [57,58] did not allow us to detect the rates of net AA uptake and release or AA synthesis and catabolism and, therefore, gain a better understanding of the origins of AA changes during FOLFOX or XELOX therapy. Thus, as discussed above, the mechanisms described that underlie AA changes should be considered as working hypotheses, at present. However, it is a matter of fact that our study found three-month CMT to be associated with abnormal plasma AA changes whose degree was dependent on the CMT type adopted. This may prompt physicians who use the mentioned drugs on resected patients to place special emphasis on their metabolic side effects.

Another limitation is the absence of markers of lipid hyper oxidation before surgery. These markers would have provided useful information about the contribution of cancer/surgery to oxidative stress at CMT initiation. A further restriction is the absence of markers of nitrogen and glucose oxidation during pre-surgery, surgery, and CMT. 

To interpret the results from the study, we inferred diet adequacy indirectly by considering the maintenance over time of body weight and serum creatinine levels and asking the patients about their habitual diet. In future studies, the patients will be asked to keep at least three-day food diaries that can be used to calculate EAA intake, in addition to the other macro–micro-nutrients.

Measures of skeletal muscle mass, strength, evaluation of patients’ physical function, and their possible correlations with both muscle AA and MDA fluxes would have provided better information on CMT-induced muscle toxicity, in this way strengthening the discussion. Finally, mutational status of the cancer [45] and its possible correlation with PAALs and MDA levels in the pre-surgery period might be controlled.

Future investigations will address the changes in PAALs and MDA separately in male and female patients.

## 4. Materials and Methods

### 4.1. Population

This observational, prospective cohort study was carried out on fourteen ambulatory patients with CRC, who were scheduled to receive FOLFOX therapy (n = 9) or XELOX therapy (n = 5) as per the standard of care. The patients were enrolled in the Medical Oncology Unit, Fondazione IRCCS Policlinico San Matteo, Pavia (Italy) from March 2017 to October 2017. Informed consent for inclusion was given by all patients before they were enrolled in the study that was conducted in accordance with the Declaration of Helsinki. The protocol followed was approved by the Ethics Committee of Policlinico San Matteo (Pavia, Italy) (Project identification code: P-20130028952, protocol n. 20140004967, 17 November 2014).

All patients considered in this study were submitted to curative radical CRC resection. From among the nine FOLFOXs, only one patient had open surgery while the remaining eight patients had laparoscopic surgery: the operated intestinal segments were the right colon (N = 2), transverse colon (N = 1), left colon (N = 4), and rectum (N = 2). All five XELOXs had open surgery: the resected intestinal segments were the right colon (N = 2), left colon (N = 2), and rectum (N = 1). All patients were performing common physical daily life activities during the periods from post-surgery hospital discharge to initiation of treatment to three-month treatment.

As shown in Table 13, adenocarcinoma types were moderately (G2) and badly differentiated (G3) at histological examinations. 

### 4.2. Chemotherapy 

The choice of the FOLFOX or XELOX scheme occurred following patient preference for oral or infusive therapy and, less importantly, for organizational reasons of the oncological ward.

All patients were submitted to the first CMT cycle around 21 days after discharge from surgery. In brief, each cycle of XELOX therapy underwent the following procedure: day 1, infusion of OXAL (130 mg/m^2^) through a peripheral vein or by means of a central venous catheter. This step was followed by 14 days of oral capecitabine intake (1000 mg/m^2^ per os bis in days), and, in turn, by 7 days of capecitabine washout. XELOX therapy was scheduled for 8 cycles over around 6 months [54,142].

FOLFOX therapy consisted of the intravenous administration of OXAL (at a dose of 85 mg/m^2^) every 2 weeks, plus leucovorin (at a dose of 200 mg/m^2^), and 5-FU (at a dose of 400 mg/m^2^). Its subsequent dose administration (at a dose of 2400 mg/m^2^) continued for 44 h through an elastomer. In the FOLFOX scheme, all drugs were administered intravenously. The schedule of the FOLFOX therapy was the following: 12 cycles over (around) 6 months.

### 4.3. Plasma AA and MDA Measures

Detection of plasma AAs and MDA was performed in peripheral venous blood that was drawn from the antecubital vein in fasting patients between 8 and 9 am. The following time points were considered: (i) before the first CMT cycle (TA), (ii) at 1 month (TB), and (iii) at 3 months of treatment (TC). Blood samples were drawn at each time point (A, B, and C), before the infusion of FOLFOX and XELOX.

#### 4.3.1. AA Measurements

Immediately after withdrawal, blood samples were delivered to the laboratory. To measure plasma AA levels, blood was heparinized and centrifuged at 800× *g*, for 15 min. 

An AminoQuant II amino acid analyzer, based on the HP 1090 HPLC platform, was used to detect the AAs and measure their concentration. Primary and secondary AAs were derivatized (by means of a fully automated precolumn derivatization system) with ortho-phthalaldehyde (OPA) and 9-fluorenyl-methyl-chloroformate (FMOC), respectively. Analysis was performed on 1 µL of sample and separation of AAs was achieved by applying the solvent gradient suggested by the manufacturer. To detect both primary and secondary AAs, absorbance was measured simultaneously at 338 and 262 nm. The concentration of plasma AAs was expressed as µmol/L. All measurements were carried out in triplicate by the same laboratory and the final value was the mean of three measurements. To check the precision of the method, a variety of parameters were determined. Relative standard deviation (RSD) was 1.13% and reliability (bias) was 10.37%. The limit of detection (LOD) was 0.0016 µmol/L and the lower limit of quantitation (LOQ) was 0.0055 µmol/L. With regard to measurements in triplicate, the intra-day and inter-day variabilities (RSDs) were 3.21% and 4.77%, respectively.

The concentrations of the following AAs were measured: (1) TAAs; (2) EAAs, leucine, valine, isoleucine (which constitute the BCAAs), lysine, threonine, phenylalanine, methionine, and tryptophan; (3) NEAAs, aspartic acid, glutamic acid, histidine, asparagine, serine, glutamine, arginine, citrulline, glycine, alanine, cysteine, ornithine, and tyrosine.

The muscle catabolism/anabolism ratio was determined by measuring the tyrosine/leucine ratio [69]. The muscle mitochondria BCAA utilization was calculated by evaluating the increase in plasma glutamine and alanine levels in relation to their baseline values [(glutamine + alanine)/BCAA ratio] [61].

Possible reductions in PAALs during CMT (in comparison to baseline values), were indicated as negative changes. By contrast, possible increases or no reductions were indicated as positive changes.

#### 4.3.2. Procedure of MDA Measurement

The level of oxidative stress was measured by determining the concentration (expressed in μmol/L) of MDA, a naturally occurring marker of lipid peroxidation. MDA was measured using the “Cayman’s TBARS Assay Kit” (Cayman Chemical Company, Ann Arbor, MI, USA) on a microplate spectrophotometer (BioTek ELx800, BioTek Instruments, Winooski, VT, USA), according to the manufacturer’s instructions (the normal range considered was: 1.86–3.94 μmol/L). 

All measurements were carried out in triplicate. The final value adopted was the mean of three independent determinations.

### 4.4. Demographic, Anthropometric, Bio-Humoral Variable Measures

Table 1 describes the baseline demographic and anthropometric characteristics and the bio-humoral variables of the enrolled patients stratified by CMT types.

Fifteen healthy, non-smoking subjects matched for age, sex, and body mass index (BMI, kg/m^2^), who performed normal daily physical activities, served as the CTR. For this CTR group, peripheral venous blood samples were only drawn to measure AAs after overnight fasting at TA.

### 4.5. Statistical Analysis

Descriptive statistics are reported as mean ± SD for continuous variables and as numbers (N) and percentage (%) for discrete variables. The normality of the distribution of each variable was assessed by the Shapiro–Wilk test. Because several violations to the normality assumption were observed, hypothesis testing was based on non-parametric statistics: within-group comparisons for continuous variables were carried out using the Wilcoxon signed rank test and between-group comparisons were carried out using the Mann–Whitney U-test (two groups) or the Kruskal–Wallis one-way ANOVA on ranks (three groups). In the case of significant results from the Kruskal–Wallis ANOVA, post hoc analysis (Tukey–Kramer criterion) was carried out. The Chi-square test or Fisher’s exact test, as appropriate, were used to compare dichotomous variables. 

The effect of the two different treatment strategies over time (TA–TB–TC) was investigated by a two-factor analysis of variance, the first factor (between factor) being treatment (XELOX and FOLFOX) and the second factor (within factor) being time (three measurements, TA, TB, TC), with repeated measurements in the time factor. The focus of this analysis was on the interaction term (time x treatment). An overall significant interaction effect for time and treatment was followed up by post-hoc analysis (Tukey’s HSD test).

The association between pairs of variables was assessed by the Spearman’s rank correlation coefficient (Spearman r). 

All tests were two-tailed. A *p*-value < 0.05 was considered to be statistically significant. All statistical analyses were carried out using the SAS/STAT statistical package, release 9.4 (SAS Institute Inc., Cary, NC, USA).

## 5. Conclusions

This preliminary study suggests that, in resected patients with CRC, FOLFOX and XELOX therapies may change the plasma amino acid profiles in opposite directions while not significantly affecting the high pretreatment plasma marker of lipid peroxidation.

Our findings need confirmation by properly powered future research, controlling for potentially confounding factors such as diet, muscle mass, and gender, to better elucidate the mechanisms underpinning FOLFOX- and XELOX-induced plasma AA changes.

## Figures and Tables

**Table 1:** Baseline (TA) demographic, anthropometric, and bio-humoral variables in cancer population stratified for type of chemotherapeutic regimen (XELOX or FOLFOX).

Variable	TA XELOX (N = 5)	TA FOLFOX (N = 9)	*p* Value
Gender (M)	5 (100%)	6 (66.7%)	
Age (years)	63.8 ± 5.9	57.6 ± 9.2	
Body weight (kg)	70.0 ± 9.4	68.6 ± 12.5	
Body mass index (kg/m^2^)	23.6 ± 4.7	23.1 ± 4.1	
Malondialdehyde (NV 1.86–3.94 μmol/L)	6.45 ± 2.30	9.90 ± 2.92	0.037
Albumin (NV 3500–5200 mg/dL)	3634 ± 223	3448 ± 552	
Creatinine (NV: M 0.73–1.18 mg/dL; F 0.55–1.02 mg/dL)	0.91 ± 0.32	0.78 ± 0.18	
Hemoglobin (NV: M 13.2–17.3 g/dL; F 11.7–15.5 g/dL)	12.5 ± 2.1	12.0 ± 2.3	
Red blood cell count (NV: M 4.30–5.70 × 10^6^/µL; F 3.80–5.20 × 10^6^/µL)	4.38 ± 0.30	4.36 ± 0.74	
Hematocrit (NV: M 39.0–49.0%; F 35.0–45.0%)	38.7 ± 5.4	36.7 ± 6.0	
Mean corpuscular volume (NV 82.0–98.0 fL)	88.5 ± 11.8	84.7 ± 9.6	
Mean hemoglobin content (NV 27.0–32.0 pg)	28.6 ± 4.5	27.7 ± 4.0	
Total white blood cells (TWBC, NV 4.00–10.00 × 10^3^/µL)	7.04 ± 0.88	7.75 ± 3.19	
Neutrophil count (NV 2.0–8.0 × 10^3^/µL)	4.50 ± 0.73	5.38 ± 2.88	
Neutrophils % TWBC	63.8 ± 2.9	67.2 ± 9.9	
Lymphocyte count (NV 1.5–4.0 × 10^3^/µL)	1.62 ± 0.16	1.56 ± 0.76	
Lymphocytes % TWBC	23.1 ± 1.8	21.2 ± 9.7	
Neutrophil/Lymphocyte ratio	2.77 ± 0.29	3.93 ± 2.08	
Monocyte count (NV 0.1–1.0 × 10^3^/µL)	0.54 ± 0.05	0.59 ± 0.12	
Monocytes % TWBC	7.86 ± 1.62	8.52 ± 2.93	
Eosinophil count (NV 0.1–0.5 × 10^3^/µL)	0.34 ± 0.09	0.19 ± 0.17	0.029
Eosinophils % TWBC	4.64 ± 1.19	2.33 ± 1.89	0.038
Basophil count (NV 0.0–0.2 × 10^3^/µL)	0.02 ± 0.04	0.06 ± 0.05	
Basophils % TWBC	0.36 ± 0.61	0.42 ± 0.42	
Platelet count (NV 150–450 × 10^3^/µL)	366.0 ± 160.4	345.3 ± 193.9	
Total bilirubin (NV 0.20–1.10 mg/dL)	0.37 ± 0.19	0.53 ± 0.24	
ɣ-glutamyl transpeptidase (NV 11–53 mU/mL)	33.8 ± 8.8	51.4 ± 33.9	
Alanine transaminase (NV 11–34 mU/mL)	16.0 ± 4.8	20.8 ± 8.6	
Aspartate transaminase (NV 11–39 mU/mL)	10.7 ± 5.7	18.9 ± 7.7	

Data are expressed as mean ± SD. Statistical analysis: Mann–Whitney U-test for the comparison XELOX vs. FOLFOX at TA. Level of significance: *p* < 0.05. Abbreviations: TA, Time A; NVs, normal values; TWBC, total white blood cells; M, male; F, female.

**Table 2:** Baseline (TA) plasma amino acid levels (µmol/L) and their ratios in XELOXs, FOLFOXs, and control subjects.

Variable	XELOXs TA (N = 5)	FOLFOXs TA (N = 9)	Controls TA (N = 15)	*p* Value	*p* XELOXs vs. FOLFOXs	*p* XELOXs vs. Controls	*p* FOLFOXs vs. Controls
Aspartic acid	9.44 ± 2.42	7.60 ± 3.63	8.87 ± 2.83	0.64			
Glutamic acid	137.1 ± 27.9	140.1 ± 27.3	117.2 ± 41.0	0.29			
Asparagine	41.0 ± 13.0	59.0 ± 21.3	46.4 ± 15.3	0.24			
Serine	39.3 ± 13.3	45.4 ± 11.0	42.0 ± 13.9	0.67			
Glutamine	329.6 ± 160.7	246.6 ± 64.6	169.0 ± 132.7	0.040	0.70	0.057	0.19
Histidine	96.9 ± 24.2	125.0 ± 43.5	56.1 ± 54.8	0.0005	0.85	0.046	0.0008
Glycine	120.9 ± 33.6	128.8 ± 63.6	157.1 ± 48.7	0.16			
Threonine	77.7 ± 32.1	86.9 ± 23.6	102.8 ± 48.0	0.45			
Citrulline	18.1 ± 7.9	20.1 ± 5.8	N.A.	0.55			
Alanine	317.5 ± 78.8	344.0 ± 101.2	266.8 ± 76.8	0.18			
Arginine	55.3 ± 13.8	64.6 ± 19.1	64.4 ± 16.2	0.54			
Tyrosine	48.4 ± 12.6	63.6 ± 16.2	48.4 ± 17.5	0.09			
Tryptophan	48.3 ± 15.4	51.6 ± 10.8	32.7 ± 7.6	0.001	0.92	0.047	0.002
Phenylalanine	47.2 ± 7.8	53.3 ± 11.0	42.1 ± 13.2	0.053	0.75	0.47	0.046
Isoleucine	58.8 ± 13.0	63.1 ± 13.5	43.6 ± 11.3	0.005	0.95	0.09	0.008
Leucine	92.5 ± 23.6	103.3 ± 26.0	74.2 ± 18.5	0.019	0.81	0.27	0.018
Lysine	101.3 ± 43.6	126.3 ± 34.6	88.0 ± 27.7	0.045	0.41	0.79	0.034
Ornithine	106.2 ± 29.2	89.7 ± 22.5	N.A.	0.26			
Cysteine	179.9 ± 66.8	182.4 ± 77.6	148.1 ± 47.0	0.60			
Valine	174.0 ± 42.6	195.5 ± 46.4	149.6 ± 25.0	0.026	0.60	0.48	0.020
Methionine	21.5 ± 7.2	29.4 ± 7.3	21.4 ± 7.3	0.054	0.26	0.97	0.048
Phenylalanine/Tyrosine	1.01 ± 0.23	0.87 ± 0.24	0.89 ± 0.11	0.44			
Tyrosine/Leucine	0.54 ± 0.14	0.62 ± 0.09	0.66 ± 0.21	0.39			
EAAs	718.3 ± 183.3	834.4 ± 163.2	610.6 ± 146.4	0.024	0.56	0.51	0.018
BCAAs	325.4 ± 77.0	361.9 ± 83.5	267.4 ± 52.8	0.012	0.77	0.25	0.012
NEAAs	1409.2 ± 384.0	1397.5 ± 310.0	1274.8 ± 256.6	0.60			
EAAs/TAAs	0.34 ± 0.05	0.38 ± 0.03	0.32 ± 0.04	0.025	0.40	0.68	0.018
BCAAs/TAAs	0.15 ± 0.02	0.16 ± 0.02	0.14 ± 0.02	0.064			
BCAAs/EAAs	0.45 ± 0.02	0.43 ± 0.05	0.44 ± 0.05	0.61			
(Gln+Ala)/BCAAs	2.00 ± 0.56	1.66 ± 0.39	1.65 ± 0.51	0.34			
NEAAs/TAAs	0.66 ± 0.05	0.62 ± 0.03	0.68 ± 0.04	0.025	0.40	0.68	0.018
Arginine/TAAs	0.03 ± 0.00	0.03 ± 0.01	0.03 ± 0.01	0.08			

Data are expressed as mean ± SD. Statistical analysis: Kruskal–Wallis one-way ANOVA on ranks for the comparison XELOXs, FOLFOXs, and controls at TA. Post hoc comparisons: Tukey–Kramer criterion. Level of significance: *p* < 0.05. Abbreviations: TA, Time A; EAAs, essential amino acids; BCAAs, branched-chain amino acids; Gln, glutamine; Ala, alanine; TAAs, total amino acids; NEAAs, non-essential amino acids; N.A., not available.

**Table 3:** Baseline (TA) demographic, anthropometric, and bio-humoral variables in cancer population stratified for gender.

Variable	TA Males (N = 11)	TA Females (N = 3)	*p* Value
Age (years)	60.5 ± 6.6	57.0 ± 15.5	0.86
Body weight (kg)	70.9 ± 11.7	62.3 ± 6.1	0.24
Body mass index (kg/m^2^)	23.7 ± 4.5	21.5 ± 2.5	0.46
Malondialdehyde (NV 1.86–3.94 μmol/L)	8.02 ± 2.76	11.06 ± 3.83	0.16
Albumin (NV 3500–5200 mg/dL)	3581.3 ± 420.6	2862.0 ± 0.0	0.36
Creatinine (NV: M 0.73–1.18 mg/dL; F 0.55–1.02 mg/dL)	0.88 ± 0.24	0.65 ± 0.05	0.08
Hemoglobin (NV: M 13.2–17.3 g/dL; F 11.7–15.5 g/dL)	12.4 ± 2.4	11.2 ± 0.3	0.17
Red blood cell count (NV: M 4.30–5.70 × 10^6^/µL; F 3.80–5.20 × 10^6^/µL)	4.44 ± 0.63	4.08 ± 0.46	0.28
Hematocrit (NV: M 39.0–49.0%; F 35.0–45.0%)	38.3 ± 6.2	34.3 ± 1.0	0.13
Mean corpuscular volume (NV 82.0–98.0 fL)	86.5 ± 11.1	84.5 ± 6.9	0.55
Mean hemoglobin content (NV 27.0–32.0 pg)	28.1 ± 4.4	27.7 ± 3.0	0.88
Total white blood cells (TWBC, NV 4.00–10.00 × 10^3^/µL)	7.20 ± 2.10	8.58 ± 4.32	0.77
Neutrophil count (NV 2.0–8.0 × 10^3^/µL)	4.73 ± 1.78	6.30 ± 4.09	0.77
Neutrophils % TWBC	64.7 ± 7.4	70.6 ± 10.6	0.46
Lymphocyte count (NV 1.5–4.0 × 10^3^/µL)	1.60 ± 0.61	1.50 ± 0.69	0.80
Lymphocytes % TWBC	22.9 ± 7.8	18.2 ± 7.7	0.46
Neutrophil/Lymphocyte ratio	3.28 ± 1.71	4.40 ± 1.88	0.45
Monocyte count (NV 0.1–1.0 × 10^3^/µL)	0.57 ± 0.11	0.57 ± 0.06	1.00
Monocytes % TWBC	8.36 ± 2.30	8.00 ± 3.70	0.86
Eosinophil count (NV 0.1–0.5 × 10^3^/µL)	0.27 ± 0.16	0.13 ± 0.12	0.26
Eosinophils % TWBC	3.34 ± 1.97	2.50 ± 2.33	0.60
Basophil count (NV 0.0–0.2 × 10^3^/µL)	0.04 ± 0.05	0.07 ± 0.06	0.77
Basophils % TWBC	0.35 ± 0.48	0.57 ± 0.51	0.54
Platelet count (NV 150–450 × 10^3^/µL)	334.5 ± 172.1	419.7 ± 214.2	0.66
Total bilirubin (NV 0.20–1.10 mg/dL)	0.48 ± 0.26	0.46 ± 0.10	0.79
ɣ-glutamyl transpeptidase (NV 11–53 mU/mL)	48.4 ± 30.7	24.5 ± 10.6	0.23
Alanine transaminase (NV 11–34 mU/mL)	19.3 ± 8.4	18.3 ± 4.7	1.00
Aspartate transaminase (NV 11–39 mU/mL)	16.3 ± 9.1	16.0 ± 1.4	1.00

Data are expressed as mean ± SD. Statistical analysis: Mann–Whitney U-test for the comparison males vs. females at TA. Level of significance: *p* < 0.05. Abbreviations: TA, Time A; NVs, normal values; TWBC, total white blood cells; M, male; F, female.

**Table 4:** Baseline (TA) plasma amino acid levels (µmol/L) and their ratios in the patient population stratified for gender.

Variable	TA Males (N = 11)	TA Females (N = 3)	*p* Value
Aspartic acid	8.29 ± 2.25	8.14 ± 6.68	0.55
Glutamic acid	137.0 ± 24.7	146.5 ± 37.5	0.55
Asparagine	49.2 ± 18.7	65.1 ± 25.4	0.29
Serine	39.6 ± 10.1	56.4 ± 7.2	0.022
Glutamine	278.3 ± 122.4	268.7 ± 66.2	1.00
Histidine	101.7 ± 32.4	163.7 ± 15.2	0.011
Glycine	110.4 ± 32.0	183.0 ± 84.6	0.09
Threonine	77.9 ± 21.6	104.9 ± 35.1	0.37
Citrulline	19.2 ± 6.5	19.9 ± 7.4	0.77
Alanine	318.4 ± 89.3	393.7 ± 89.3	0.17
Arginine	60.9 ± 17.1	62.6 ± 22.9	1.00
Tyrosine	56.9 ± 16.8	62.9 ± 17.1	0.55
Tryptophan	49.7 ± 12.8	53.2 ± 10.8	0.88
Phenylalanine	48.5 ± 9.5	60.8 ± 5.3	0.09
Isoleucine	59.1 ± 13.6	70.7 ± 4.7	0.17
Leucine	96.9 ± 26.4	108.9 ± 18.9	0.37
Lysine	110.5 ± 38.7	142.4 ± 30.6	0.29
Ornithine	92.7 ± 26.3	106.3 ± 22.1	0.46
Cysteine	161.7 ± 48.4	254.1 ± 106.1	0.022
Valine	186.4 ± 48.1	193.0 ± 36.5	0.66
Methionine	24.9 ± 7.1	32.7 ± 9.6	0.29
Phenylalanine/Tyrosine	0.90 ± 0.25	1.00 ± 0.23	0.55
Tyrosine/Leucine	0.60 ± 0.12	0.57 ± 0.06	0.77
EAAs	755.5 ± 174.6	930.3 ± 83.6	0.13
BCAAs	342.4 ± 86.5	372.6 ± 58.0	0.46
NEAAs	1338.5 ± 307.4	1633.4 ± 323.5	0.13
EAAs/TAAs	0.36 ± 0.04	0.37 ± 0.06	0.88
BCAAs/TAAs	0.16 ± 0.02	0.15 ± 0.03	0.46
BCAAs/EAAs	0.45 ± 0.04	0.40 ± 0.03	0.060
(Gln+Ala)/BCAAs	1.76 ± 0.43	1.84 ± 0.66	0.88
NEAAs/TAAs	0.64 ± 0.04	0.63 ± 0.06	0.88
Arginine/TAAs	0.03 ± 0.01	0.02 ± 0.01	0.46

Data are expressed as mean ± SD. Statistical analysis: Mann–Whitney U-test for the comparison males vs. females at TA. Level of significance: *p* < 0.05. Abbreviations: TA, Time A; EAAs, essential amino acids; BCAAs, branched-chain amino acids; Gln, glutamine; Ala, alanine; TAAs, total amino acids; NEAAs, non-essential amino acids.

**Table 5:** Baseline (TA) differences in amino acid/creatinine ratios between males and females.

Variable	TA Males (N = 11)	TA Females (N = 3)	*p* Value
Aspartic acid/creatinine	10.0 ± 3.4	12.5 ± 10.6	0.66
Glutamic acid/creatinine	167.6 ± 59.3	226.2 ± 55.6	0.17
Asparagine/creatinine	62.8 ± 36.2	99.4 ± 33.0	0.17
Serine/creatinine	49.2 ± 21.3	87.9 ± 16.6	0.038
Glutamine/creatinine	339.8 ± 168.3	419.5 ± 121.7	0.46
Histidine/creatinine	125.7 ± 56.8	253.2 ± 16.5	0.011
Glycine/creatinine	132.1 ± 42.0	285.7 ± 142.0	0.022
Threonine/creatinine	96.7 ± 42.0	165.2 ± 63.5	0.038
Citrulline/creatinine	21.9 ± 4.5	31.4 ± 13.1	0.29
Alanine/creatinine	389.6 ± 157.9	611.8 ± 155.4	0.060
Arginine/creatinine	75.7 ± 33.9	97.3 ± 38.4	0.23
Tyrosine/creatinine	71.7 ± 34.2	97.9 ± 29.9	0.29
Tryptophan/creatinine	61.6 ± 27.9	82.1 ± 14.2	0.13
Phenylalanine/creatinine	59.5 ± 21.6	94.7 ± 14.2	0.022
Isoleucine/creatinine	72.9 ± 29.8	109.9 ± 14.1	0.09
Leucine/creatinine	121.4 ± 58.7	169.0 ± 32.7	0.13
Lysine/creatinine	142.7 ± 86.8	219.9 ± 44.8	0.17
Ornithine/creatinine	116.2 ± 61.6	166.4 ± 43.8	0.09
Cysteine/creatinine	191.7 ± 50.6	398.2 ± 182.7	0.011
Valine/creatinine	232.9 ± 110.2	300.4 ± 68.2	0.13
Methionine/creatinine	24.9 ± 7.1	32.7 ± 9.6	0.29
EAAs/creatinine	944.7 ± 429.7	1445.1 ± 187.1	0.09
BCAAs/creatinine	427.2 ± 197.9	579.4 ± 110.8	0.13
NEAAs/creatinine	1634.4 ± 576.6	2543.6 ± 604.2	0.060
TAAs/creatinine	2579.1 ± 989.3	3988.8 ± 615.7	0.09

Data are expressed as mean ± SD. Statistical analysis: Mann–Whitney U-test for the comparison males vs. females at TA. Level of significance: *p* < 0.05. Abbreviations: TA, Time A; EAAs, essential amino acids; BCAAs, branched-chain amino acids; NEAAs, non-essential amino acids; TAAs, total amino acids.

**Table 6:** Time courses (Time A → Time B → Time C) of body weight, body mass index, and creatinine in XELOXs (N = 5) and FOLFOXs (N = 9).

Variable	TA XELOX	TA FOLFOX	TB XELOX	TB FOLFOX	TC XELOX	TC FOLFOX	*p*-Time	*p*-Group	*p*-Group x Time Interaction
Body weight (kg)	70.0 ± 9.4	68.6 ± 12.5	71.6 ± 8.6	68.0 ± 13.1	72.0 ± 8.7	69.0 ± 12.8	0.10	0.69	0.15
Body mass index (kg/m^2^)	23.6 ± 4.7	23.1 ± 4.1	24.1 ± 4.4	22.9 ± 4.3	24.2 ± 4.6	23.2 ± 4.1	0.11	0.71	0.15
Creatinine (mg/dL)	0.91 ± 0.32	0.78 ± 0.18	0.89 ± 0.22	0.78 ± 0.22	0.97 ± 0.21	0.79 ± 0.15	0.38	0.24	0.54

Data are expressed as mean ± SD. Statistical analysis: two-factor analysis of variance, the first factor being treatment (between factors, two groups: XELOX and FOLFOX) and the second factor being time (within factors, three measurements: TA, TB, and TC), with repeated measurements in the time factor. *p*-time: *p*-value for time effect in repeated measures ANOVA; *p*-group: *p*-value for treatment effect in repeated measures ANOVA; *p*-group x time interaction: *p*-value for interaction term treatment x time in repeated measures ANOVA. Level of significance: *p* < 0.05. Abbreviations: TA, Time A; TB, Time B; TC, Time C.

**Table 7:** Time courses (Time A → Time B → Time C) of plasma amino acid levels (µmol/L) in XELOXs (N = 5) and FOLFOXs (N = 9).

Variable	TA XELOXs	TA FOLFOXs	TB XELOXs	TB FOLFOXs	TC XELOXs	TC FOLFOXs	*p*-Time	*p*-Group	*p*-Group x Time Interaction
Aspartic acid	9.44 ± 2.42	8.21 ± 3.95	9.98 ± 1.34	6.85 ± 3.51	10.18 ± 3.29	6.51 ± 2.75	0.84	0.09	0.37
Glutamic acid	137.1 ± 27.9	147.1 ± 31.4	160.6 ± 34.5	148.8 ± 25.5	164.5 ± 46.8	136.2 ± 29.6	0.50	0.45	0.22
Asparagine	41.0 ± 13.0	55.7 ± 19.0	49.9 ± 17.7	52.5 ± 16.2	62.5 ± 25.2	47.8 ± 14.4	0.54	0.90	0.070
Serine	39.3 ± 13.3	46.0 ± 11.2	47.2 ± 17.5	37.2 ± 10.0	48.3 ± 12.8	33.7 ± 7.9	0.90	0.25	0.019
Glutamine	329.6 ± 160.7	275.6 ± 111.4	423.9 ± 174.2	264.6 ± 135.9	431.4 ± 149.7	265.5 ± 130.7	0.23	0.10	0.11
Histidine	96.9 ± 24.2	126.9 ± 42.3	128.4 ± 62.1	137.7 ± 32.8	132.4 ± 44.5	125.0 ± 40.8	0.19	0.58	0.31
Glycine	120.9 ± 33.6	136.3 ± 62.1	155.0 ± 48.6	143.2 ± 42.0	150.2 ± 28.6	138.0 ± 46.9	0.29	0.90	0.50
Threonine	77.7 ± 32.1	88.5 ± 24.2	88.8 ± 30.7	92.2 ± 38.9	94.8 ± 23.2	75.2 ± 17.7	0.64	0.89	0.16
Citrulline	18.1 ± 7.9	21.4 ± 5.8	23.0 ± 7.8	17.4 ± 8.0	26.2 ± 12.0	17.1 ± 8.3	0.67	0.33	0.029
Alanine	317.5 ± 78.8	371.5 ± 98.1	421.0 ± 134.9	335.3 ± 91.8	469.5 ± 196.5	287.4 ± 78.4	0.69	0.062	0.045
Arginine	55.3 ± 13.8	65.1 ± 19.7	65.2 ± 28.5	51.2 ± 17.9	50.5 ± 12.3	38.8 ± 16.5	0.016	0.54	0.068
Tyrosine	48.4 ± 12.6	64.3 ± 16.0	57.1 ± 17.5	48.4 ± 12.9	75.1 ± 29.3	49.8 ± 18.2	0.35	0.36	0.016
Tryptophan	48.3 ± 15.4	55.4 ± 12.2	62.7 ± 15.5	45.1 ± 9.3	65.5 ± 13.2	43.0 ± 9.2	0.85	0.023	0.007
Phenylalanine	47.2 ± 7.8	56.9 ± 11.3	60.0 ± 6.6	50.1 ± 12.1	74.6 ± 25.9	51.3 ± 12.9	0.11	0.12	0.013
Isoleucine	58.8 ± 13.0	67.9 ± 16.0	62.4 ± 16.8	53.4 ± 19.1	76.7 ± 30.0	44.9 ± 12.4	0.71	0.13	0.017
Leucine	92.5 ± 23.6	110.8 ± 31.7	93.7 ± 33.6	79.1 ± 26.5	116.4 ± 53.0	59.9 ± 19.0	0.25	0.21	0.003
Lysine	101.3 ± 43.6	127.9 ± 34.9	136.7 ± 77.7	109.4 ± 31.6	145.7 ± 83.1	80.2 ± 17.5	0.74	0.31	0.010
Ornithine	106.2 ± 29.2	97.7 ± 21.4	129.7 ± 40.6	80.7 ± 18.9	137.4 ± 40.6	69.3 ± 13.1	0.93	0.002	0.006
Cysteine	179.9 ± 66.8	198.4 ± 78.6	197.6 ± 46.6	213.0 ± 72.5	193.0 ± 47.6	196.8 ± 65.8	0.65	0.70	0.90
Valine	174.0 ± 42.6	208.1 ± 51.4	186.6 ± 60.8	160.5 ± 50.0	226.0 ± 98.9	122.6 ± 27.2	0.55	0.18	0.003
Methionine	21.5 ± 7.2	29.9 ± 7.4	29.4 ± 9.0	27.1 ± 9.0	35.9 ± 14.1	26.1 ± 8.6	0.28	0.73	0.034
Tyrosine/Leucine	0.54 ± 0.14	0.60 ± 0.12	0.64 ± 0.17	0.65 ± 0.14	0.70 ± 0.26	0.87 ± 0.37	0.032	0.39	0.58
Phenylalanine/Tyrosine	1.01 ± 0.23	0.93 ± 0.25	1.10 ± 0.19	1.09 ± 0.36	1.01 ± 0.17	1.10 ± 0.30	0.08	1.00	0.30
TAAs	2128 ± 518	2366 ± 498	2596 ± 743	2160 ± 504	2791 ± 750	1920 ± 396	0.78	0.10	0.034
EAAs	718 ± 183	872 ± 172	849 ± 289	755 ± 179	968 ± 351	628 ± 105	1.00	0.26	0.009
BCAAs	325.4 ± 77.0	386.8 ± 97.0	342.7 ± 110.7	293.0 ± 93.0	419.1 ± 180.5	227.4 ± 56.4	0.48	0.17	0.004
(Gln+Ala)/BCAAs	2.00 ± 0.56	1.66 ± 0.39	2.52 ± 0.66	2.08 ± 0.85	2.26 ± 0.46	2.26 ± 0.53	0.012	0.38	0.35
NEAAs	1409 ± 384	1493 ± 359	1747 ± 482	1406 ± 363	1823 ± 426	1292 ± 323	0.59	0.10	0.07

Data are expressed as mean ± SD. Statistical analysis: two-factor analysis of variance, the first factor being treatment (between factors, two groups: XELOX and FOLFOX) and the second factor being time (within factors, three measurements: TA, TB and TC), with repeated measurements in the time factor. *p*-time: *p*-value for time effect in repeated measures ANOVA; *p*-group: *p*-value for treatment effect in repeated measures ANOVA; *p*-group x time interaction: *p*-value for interaction term treatment x time in repeated measures ANOVA. Level of significance: *p* < 0.05. Abbreviations: TA, Time A; TB, Time B; TC, Time C; EAAs, essential amino acids; BCAAs, branched-chain amino acids; TAAs, total amino acids; Gln, glutamine; Ala, alanine; NEAAs, non-essential amino acids.

**Table 8:** Time courses (Time A → Time B → Time C) of plasma amino acid levels (µmol/L) and plasma malondialdehyde (MDA; μmol/L) in FOLFOXs (N = 9) stratified for gender.

Variable	TA Males (N = 6)	TA Females (N = 3)	TB Males (N = 6)	TB Females (N = 3)	TC Males (N = 6)	TC Females (N = 3)	*p*-Time	*p*-Gender	*p*-Gender x Time Interaction
Aspartic acid	7.42 ± 1.92	8.14 ± 6.68	6.59 ± 3.88	4.96 ± 3.21	6.42 ± 3.71	6.23 ± 1.36	0.33	0.78	0.33
Glutamic acid	138.9 ± 26.7	146.5 ± 37.5	149.4 ± 30.2	131.8 ± 4.5	136.0 ± 40.7	132.1 ± 8.7	0.65	0.63	0.39
Asparagine	50.2 ± 17.3	65.1 ± 25.4	49.0 ± 14.3	40.2 ± 3.8	46.5 ± 11.7	48.3 ± 23.3	0.16	0.94	0.08
Serine	38.6 ± 8.2	56.4 ± 7.2	34.8 ± 8.0	34.7 ± 8.2	30.7 ± 7.0	36.1 ± 9.1	0.001	0.14	0.038
Glutamine	231.2 ± 74.0	268.7 ± 66.2	225.1 ± 72.5	196.7 ± 72.8	243.5 ± 80.9	202.4 ± 57.1	0.43	0.73	0.22
Histidine	108.4 ± 44.0	163.7 ± 15.2	138.9 ± 41.2	137.2 ± 29.1	119.4 ± 52.1	135.8 ± 31.2	0.69	0.40	0.12
Glycine	104.3 ± 33.7	183.0 ± 84.6	138.8 ± 47.4	139.6 ± 43.2	127.7 ± 53.2	148.2 ± 49.9	0.92	0.36	0.056
Threonine	76.0 ± 10.5	104.9 ± 35.1	87.9 ± 42.4	80.3 ± 40.8	70.7 ± 17.2	76.9 ± 21.2	0.30	0.68	0.19
Citrulline	21.3 ± 5.6	19.9 ± 7.4	17.2 ± 8.5	10.9 ± 3.6	18.6 ± 8.2	11.6 ± 6.8	0.10	0.20	0.25
Alanine	338.2 ± 105.0	393.7 ± 89.3	318.7 ± 82.2	253.5 ± 8.4	290.4 ± 97.4	260.3 ± 47.5	0.13	0.53	0.20
Arginine	62.6 ± 20.0	62.6 ± 22.9	51.1 ± 22.3	46.6 ± 15.5	34.7 ± 13.1	41.3 ± 24.7	0.009	0.99	0.58
Tyrosine	65.4 ± 19.0	62.9 ± 17.1	48.4 ± 8.1	41.1 ± 20.1	52.5 ± 20.4	47.9 ± 20.8	0.08	0.55	0.80
Tryptophan	53.0 ± 11.7	53.2 ± 10.8	43.8 ± 7.2	36.6 ± 7.5	45.5 ± 11.0	36.6 ± 1.0	0.046	0.17	0.44
Phenylalanine	51.5 ± 11.5	60.8 ± 5.3	49.5 ± 15.3	45.3 ± 7.7	51.0 ± 17.0	51.6 ± 9.3	0.34	0.89	0.32
Isoleucine	61.1 ± 16.4	70.7 ± 4.7	51.3 ± 14.2	32.5 ± 6.8	48.7 ± 13.8	40.0 ± 12.5	0.004	0.23	0.011
Leucine	102.1 ± 33.5	108.9 ± 18.9	76.3 ± 17.6	59.8 ± 35.4	60.1 ± 20.4	61.1 ± 24.3	0.00049	0.71	0.15
Lysine	117.1 ± 40.4	142.4 ± 30.6	107.0 ± 14.7	104.1 ± 57.8	76.6 ± 14.7	81.5 ± 25.8	0.009	0.64	0.54
Ornithine	86.7 ± 15.7	106.3 ± 22.1	78.2 ± 11.4	65.5 ± 23.7	72.1 ± 13.3	60.7 ± 11.4	0.006	0.61	0.052
Cysteine	151.3 ± 21.2	254.1 ± 106.1	208.1 ± 88.8	199.8 ± 64.9	178.2 ± 68.9	212.4 ± 74.4	0.89	0.40	0.08
Valine	203.6 ± 57.2	193.0 ± 36.5	156.9 ± 37.8	117.5 ± 54.5	126.5 ± 33.5	115.3 ± 24.3	0.0009	0.32	0.18
Methionine	27.7 ± 7.0	32.7 ± 9.6	26.7 ± 7.9	19.9 ± 9.2	28.1 ± 8.7	23.7 ± 11.2	0.32	0.52	0.10
EAAs	800.5 ± 194.9	930.3 ± 83.6	738.3 ± 138.8	633.3 ± 232.3	626.6 ± 106.7	622.6 ± 145.5	0.012	0.87	0.12
BCAAs	366.7 ± 106.5	372.6 ± 58.0	284.5 ± 68.4	209.8 ± 96.3	235.3 ± 66.0	216.4 ± 60.5	0.0007	0.40	0.10
NEAAs	1302 ± 271	1633 ± 324	1331 ± 331	1170 ± 208	1243 ± 357	1210 ± 157	0.21	0.93	0.12
TAAs	2103 ± 464	2564 ± 267	2069 ± 462	1803 ± 348	1870 ± 449	1832 ± 271	0.07	1.00	0.11
MDA	9.64 ± 2.73	11.06 ± 3.83	8.61 ± 0.89	10.00 ± 5.92	7.18 ± 0.53	9.39 ± 4.51	0.041	0.39	0.82

Data are expressed as mean ± SD. Statistical analysis: two-factor analysis of variance, the first factor being gender (between factors, two levels: males and females) and the second factor being time (within factors, three measurements: TA, TB and TC), with repeated measurements in the time factor. *p*-time: *p*-value for time effect in repeated measures ANOVA; *p*-gender: *p*-value for gender effect in repeated measures ANOVA; *p*-gender x time interaction: *p*-value for interaction term gender x time in repeated measures ANOVA. Level of significance: *p* < 0.05. Abbreviations: TA, Time A; TB, Time B; TC, Time C; EAAs, essential amino acids; BCAAs, branched-chain amino acids; NEAAs, non-essential amino acids; TAAs, total amino acids; malondialdehyde, MDA.

**Table 9:** Time course (Time A → Time B → Time C) of plasma malondialdehyde (MDA) in XELOXs (N = 5) and FOLFOXs (N = 9).

Variable	TA XELOX	TA FOLFOX	TB XELOX	TB FOLFOX	TC XELOX	TC FOLFOX	*p*-Time	*p*-Group	*p*-Group x Time Interaction
MDA (NV 1.86–3.94 μmol/L)	6.45 ± 2.30	9.90 ± 2.92	6.63 ± 0.56	8.31 ± 3.34	6.09 ± 2.11	8.00 ± 2.52	0.18	0.09	0.29

Data are expressed as mean ± SD. Statistical analysis: two-factor analysis of variance, the first factor being treatment (between factors, two groups: XELOX and FOLFOX) and the second factor being time (within factors, three measurements: TA, TB and TC), with repeated measurements in the time factor. *p*-time: *p*-value for time effect in repeated measures ANOVA; *p*-group: *p*-value for treatment effect in repeated measures ANOVA; *p*-group x time interaction: *p* -value for interaction term treatment x time in repeated measures ANOVA. Level of significance: *p* < 0.05. Abbreviations: TA, Time A; TB, Time B; TC, Time C; MDA, malondialdehyde; NVs, normal values.

**Table 10:** Plasma amino acid levels (µmol/L) at the end of three-month treatment (Time C) compared to baseline values (Time A) in XELOXs (N = 5) and FOLFOXs (N = 9).

Variable	TA XELOX	TC XELOX	TA FOLFOX	TC FOLFOX	Δ (TC−TA) XELOX	Δ (TC−TA) FOLFOX	*p* TC vs. TA XELOX	*p* TC vs. TA FOLFX
Aspartic acid	9.44 ± 2.42	10.18 ± 3.29	8.21 ± 3.95	6.51 ± 2.75	0.74 ± 3.58	−1.70 ± 3.49	1.00	0.25
Glutamic acid	137.1 ± 27.9	164.5 ± 46.8	147.1 ± 31.4	136.2 ± 29.6	27.5 ± 37.4	−10.9 ± 41.7	0.31	0.50
Asparagine	41.0 ± 13.0	62.5 ± 25.2	55.7 ± 19.0	47.8 ± 14.4	21.5 ± 27.1	−7.9 ± 11.4	0.13	0.055
Serine	39.3 ± 13.3	48.3 ± 12.8	46.0 ± 11.2	33.7 ± 7.9	9.0 ± 15.7	−12.2 ± 11.0	0.31	0.020
Glutamine	329.6 ± 160.7	431.4 ± 149.7	275.6 ± 111.4	265.5 ± 130.7	101.8 ± 101.2	−10.1 ± 80.9	0.063	0.82
Histidine	96.9 ± 24.2	132.4 ± 44.5	126.9 ± 42.3	125.0 ± 40.8	35.5 ± 43.3	−1.9 ± 41.6	0.063	0.91
Glycine	120.9 ± 33.6	150.2 ± 28.6	136.3 ± 62.1	138.0 ± 46.9	29.3 ± 37.4	1.8 ± 47.5	0.063	0.91
Threonine	77.7 ± 32.1	94.8 ± 23.2	88.5 ± 24.2	75.2 ± 17.7	17.1 ± 31.0	−13.3 ± 19.5	0.31	0.10
Citrulline	18.1 ± 7.9	26.2 ± 12.0	21.4 ± 5.8	17.1 ± 8.3	8.2 ± 9.7	−4.3 ± 7.2	0.13	0.10
Alanine	317.5 ± 78.8	469.5 ± 196.5	371.5 ± 98.1	287.4 ± 78.4	151.9 ± 246.5	−84.0 ± 133.1	0.063	0.13
Arginine	55.3 ± 13.8	50.5 ± 12.3	65.1 ± 19.7	38.8 ± 16.5	−4.7 ± 14.1	−26.2 ± 17.9	0.44	0.004
Tyrosine	48.4 ± 12.6	75.1 ± 29.3	64.3 ± 16.0	49.8 ± 18.2	26.7 ± 38.9	−14.5 ± 23.2	0.19	0.10
Tryptophan	48.3 ± 15.4	65.5 ± 13.2	55.4 ± 12.2	43.0 ± 9.2	17.2 ± 24.5	−12.4 ± 13.7	0.31	0.039
Phenylalanine	47.2 ± 7.8	74.6 ± 25.9	56.9 ± 11.3	51.3 ± 12.9	27.4 ± 29.3	−5.6 ± 17.0	0.063	0.30
Isoleucine	58.8 ± 13.0	76.7 ± 30.0	67.9 ± 16.0	44.9 ± 12.4	17.9 ± 32.2	−23.0 ± 20.3	0.63	0.012
Leucine	92.5 ± 23.6	116.4 ± 53.0	110.8 ± 31.7	59.9 ± 19.0	23.8 ± 53.0	−51.0 ± 31.2	0.63	0.004
Lysine	101.3 ± 43.6	145.7 ± 83.1	127.9 ± 34.9	80.2 ± 17.5	44.4 ± 71.8	−47.8 ± 37.7	0.063	0.004
Ornithine	106.2 ± 29.2	137.4 ± 40.6	97.7 ± 21.4	69.3 ± 13.1	31.2 ± 50.4	−28.5 ± 26.3	0.31	0.012
Cysteine	179.9 ± 66.8	193.0 ± 47.6	198.4 ± 78.6	196.8 ± 65.8	13.1 ± 44.2	−1.6 ± 67.0	0.81	0.57
Valine	174.0 ± 42.6	226.0 ± 98.9	208.1 ± 51.4	122.6 ± 27.2	52.0 ± 108.3	−85.5 ± 46.1	0.44	0.004
Methionine	21.5 ± 7.2	35.9 ± 14.1	29.9 ± 7.4	26.1 ± 8.6	14.4 ± 16.0	−3.8 ± 11.4	0.13	0.30
TAAs	2128 ± 518	2791 ± 750	2366 ± 498	1920 ± 396	664 ± 974	−446 ± 583	0.13	0.10
EAAs	718 ± 183	968 ± 351	872 ± 172	628 ± 105	250 ± 393	−244 ± 191	0.19	0.004
BCAAs	325.4 ± 77.0	419.1 ± 180.5	386.8 ± 97.0	227.4 ± 56.4	93.7 ± 193.1	−159.5 ± 94.3	0.63	0.004
NEAAs	1409 ± 384	1823 ± 426	1493 ± 359	1292 ± 323	414 ± 585	−202 ± 411	0.063	0.25
Arginine/TAAs	0.03 ± 0.00	0.02 ± 0.00	0.03 ± 0.01	0.02 ± 0.01	−0.01 ± 0.00	−0.01 ± 0.01	0.063	0.008
BCAAs/EAAs	0.45 ± 0.02	0.43 ± 0.03	0.44 ± 0.05	0.36 ± 0.05	−0.03 ± 0.02	−0.08 ± 0.06	0.063	0.004
EAAs/TAAs	0.34 ± 0.05	0.34 ± 0.04	0.37 ± 0.03	0.33 ± 0.04	0.00 ± 0.02	−0.04 ± 0.03	1.00	0.012
BCAAs/TAAs	0.15 ± 0.02	0.15 ± 0.03	0.16 ± 0.02	0.12 ± 0.03	−0.01 ± 0.01	−0.04 ± 0.03	0.19	0.004
(Gln+Ala)/BCAAs	2.00 ± 0.56	2.26 ± 0.46	1.66 ± 0.39	2.26 ± 0.53	0.26 ± 0.31	0.60 ± 0.60	0.13	0.008
NEAAs/TAAs	0.66 ± 0.05	0.66 ± 0.04	0.63 ± 0.03	0.67 ± 0.04	−0.00 ± 0.02	0.04 ± 0.03	1.00	0.012

Data are expressed as mean ± SD. Statistical analysis: Wilcoxon signed rank test. *p* TC vs. TA XELOX: *p*-value for the comparison TC vs. TA in XELOX group; *p* TC vs. TA FOLFOX: *p*-value for the comparison TC vs. TA in FOLFOX group. Level of significance: *p* < 0.05. Abbreviations: TA, Time A; TC, Time C; EAAs, essential amino acids; BCAAs, branched-chain amino acids; Gln, glutamine; Ala, alanine; TAAs, total amino acids; NEAAs, non-essential amino acids.

**Table 11:** Some potential effects clinically relevant to CRC patients from reduced PAALs.

Reduced AAs	Body Compartments	Impaired Metabolic Activities	Increased Clinical Risks
BCAAs (leucine, valine, isoleucine)	Immune system [105]	Reduced lymphocyte synthesis of proteins, DNA, and RNA	Impaired adaptive immune response
	Bone marrow [106]	Reduced red cell production	Anemia
	Myocardium [60]	Reduced aerobic energy production	Impaired left ventricular ejection fraction
	Brain [75]	Impairment in glutamate turnover Impairment in regulation of glutamate, glutamine, and GABA Reduced protein synthesis	Increased neurotoxicity Unbalanced excitatory/inhibitory ratio
		Reduced contents in GABA, histamine, and serotonin [107]	Increased pain perception
	Gut	Development of pathogenic bacteria [84,108,109]	Gut inflammation (mucositis), increased intestinal permeability (translocation)
Tryptophan	Immune system [110]	Impaired innate immune cell activation and T cell modulation	Altered innate and adaptive immune responses
	Brain [75]	Reduced serotonin formation and serotonergic neurotransmission	Impaired cognitive and motor dysfunctions Mood alterations
Arginine	Immune system	Reduced nitric oxide generation in macrophages [111]	Reduced macrophage control and elimination of intracellular pathogens and tumor cells
		Impaired T-cell proliferation and function [92,112]	Impaired adaptive immune response Impaired wound healing
	Skeletal muscle	Impaired creatine synthesis	Impaired muscle metabolic demands
	Liver	Impaired urea cycle	Impaired maintenance of body nitrogen balance Hyperammoniemic encephalopathy [113,114]
	Brain [75]	Disruption of urea cycle Increased nitric oxide generation Nitrosative stress	Hyperammonia Degeneration of synapses and neurons
Serine	Immune system	Impaired purine synthesis in T cells [115] Impaired naive T cell activation [116]	Reduced effector T cell expansion Reduced innate immune response
Aromatic amino acids (phenylalanine, tyrosine)	Brain	Impaired synthesis of adrenergic neurotransmitters	Cognitive and motor dysfunction Mood alteration during—after CMT (chemobrain) [117,118,119]

Abbreviations: CRC, colorectal cancer; PAALs, plasma amino acid levels; AAs, amino acids; BCAAs, branched-chain amino acids; GABA, gamma-aminobutyric acid; CMT, chemotherapy.

**Table 12:** Synthesis of some putative effects of FOLFOX therapy on AA/protein metabolism leading to changes in circulating EAA levels.

**FOLFOX**	
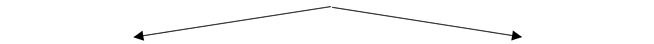	
Muscle:	Extra-muscle districts:
• Mitochondrial BCAA/EAA overutilization	• Increased BCAA/EAA uptake by:
• Increased ROS	- myocardium
• Increased proteolysis pathways	- intestine
• Decreased protein synthesis rate	- brain
• Prevalence of protein/AA hypercatabolism	- adaptive immune system
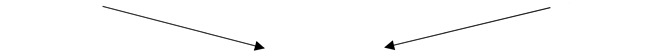	
Reductions in plasma AAs	

Abbreviations: AAs, amino acids; BCAA, branched-chain amino acid; EAA, essential amino acid; ROS, reactive oxygen species.

**Table 13:** Oncologic characteristics of colorectal cancer (CRC) surgery patients.

Patients	Differentiation Grades	TNM Staging Classification [141]
FOLFOX		
1	G2	pT3pN1a (stage III)
2	G3	pT4apN2b (stage III)
3	G2/G3	pT4apN1b (stage III)
4	G3	pT4apN2b (stage III)
5	G3	pT4apN0 (stage III)
6	G2	pT2pN2a (stage III)
7	G2	pT2pN2a (stage III)
8	G2	pT4apN0 (stage II)
9	G2/G3	pT4apN0 (stage II)
XELOX		
1	G3	pT4apN2b (stage III)
2	G2/G3	pT2pN1b (stage III)
3	G2	pT3N1b (stage III)
4	G2	pT3pN1b (stage III)
5	G2	pT4pN1a (stage III)

Abbreviations: TNM, tumor, node, metastases. Differentiation grades: G2, moderately differentiated; G3, badly differentiated.

## Data Availability

The data that support the findings of this study are available from the corresponding author upon reasonable request.
